# Unveiling the Movement of RanBP1 During the Cell Cycle and Its Interaction with a Cyclin-Dependent Kinase (CDK) in Plants

**DOI:** 10.3390/ijms26010046

**Published:** 2024-12-24

**Authors:** Vanessa Thomé, Pedro B. Ferreira, Greice Lubini, Fernanda M. Nogueira, Edward J. Strini, Vitor F. Pinoti, Joelma O. Cruz, Juca A. B. San Martin, Andréa C. Quiapim, Luis L. P. daSilva, Maria Helena S. Goldman

**Affiliations:** 1 Departamento de Biologia, Faculdade de Filosofia, Ciências e Letras de Ribeirão Preto, Universidade de São Paulo, Ribeirão Preto 14040-901, SP, Brazil; vanessa.thome@usp.br (V.T.); pedrobf@alumni.usp.br (P.B.F.); greicelubini@gmail.com (G.L.); edstrini@gmail.com (E.J.S.); vitorpinoti@usp.br (V.F.P.); joelmaioliveira20@gmail.com (J.O.C.); jsanmartin@darwin.edu.ar (J.A.B.S.M.); andreacqusp@hotmail.com (A.C.Q.); 2 PPG Genética, Faculdade de Medicina de Ribeirão Preto, Universidade de São Paulo, Ribeirão Preto 14001-970, SP, Brazil; 3 Departamento de Biologia Celular e Molecular e Bioagentes Patogênicos, Faculdade de Medicina de Ribeirão Preto, Universidade de São Paulo, Ribeirão Preto 14049-900, SP, Brazil; lldasilva@fmrp.usp.br

**Keywords:** CDKG;2, mitotic spindle, nuclear arrest, mitosis onset, RanGTPase system, protein interaction, *Nicotiana tabacum*, nuclear export signal

## Abstract

In the *Nicotiana tabacum* flower development study, we identified SCI1 (Stigma/style Cell-cycle Inhibitor 1), a regulator of cell proliferation. SCI1 interacts with NtCDKG;2 (*N. tabacum* Cyclin-Dependent Kinase G;2), a homolog of human CDK11, which is responsible for RanGTP-dependent microtubule stabilization, regulating spindle assembly rate. In a Y2H screening of a cDNA library using NtCDKG;2 as bait, a RanBP1 (Ran-Binding Protein 1) was revealed as its interaction partner. RanBP1 is an essential regulatory protein of the RanGTPase system, contributing to the formation of the Ran gradient, which modulates different important cellular processes. RanBP1 is crucial in the nuclear import/export machinery during interphase and spindle checkpoint formation during cell division. These processes are well studied in animals, but very little is known about them in plants. We confirmed NtCDKG;2 and NtRanBP1 interaction by pairwise Y2H and characterized the localization of both proteins during plant cell division. We demonstrated the presence of NtRanBP1 in the cytoplasm during interphase and its nuclear arrest at mitosis onset. Meanwhile, we showed that NtCDKG;2 is localized in the mitotic spindle during cell division, indicating an analogous function to the human CDK11. We propose that the phosphorylation of the nuclear export signal at RanBP1 by NtCDKG;2 may be responsible for the reported nuclear arrest.

## 1. Introduction

Cell proliferation is a crucial biological process for plants, as their organs’ development occurs continuously throughout their life cycle [1]. Our previous studies showed that SCI1 (Stigma/style Cell-cycle Inhibitor 1) regulates the stigma/style final size in *Nicotiana tabacum* and *Arabidopsis thaliana* [2,3]. It has been hypothesized that SCI1 regulates the cell cycle by inhibiting cyclin-CDK complexes [2,4]. In the search for SCI1 interaction partners in *N. tabacum* using pulldown assays, a CDK homologous to CDKG;2 from *A. thaliana* was identified [5]. The interaction between SCI1 and NtCDKG;2 was further confirmed by BiFC (Bimolecular Fluorescent Complementation) [5].

In eukaryotes, phosphorylations carried out by kinases are one of the most essential forms of post-translational regulation [6]. In plants, there is a considerably greater quantity of kinases than that found in other eukaryotes: while they present between 600 and 2500 of these proteins, *Drosophila* sp. and *Homo sapiens* have 239 and 528 kinases, respectively [7,8]. In plants, CDKs are divided into seven classes, CDKA to CDKG, based on their cyclin-binding motif sequences [9,10]. These proteins act in several aspects of the cell cycle, such as checkpoints, entry into mitosis, signaling for DNA repair, RNA metabolism, and regulation of other CDKs [1,11,12].

The CDKG class has two members in the Arabidopsis genome: CDKG;1 and CDKG;2 [13]. CDKG;1 interacts with the splicing factor RS2Z33 and regulates pre-mRNA splicing of the *CalS5* (*CALLOSE SYNTHASE 5*) gene [14]. Furthermore, this CDK participates in synapsis and chromosome recombination processes during male gametogenesis, in complex with Cyclin L1 [15]. CDKG;2 regulates early vegetative development and flowering [16,17]. Also, studies have shown the participation of CDKG;2 in splicing [5,18,19]. However, its participation in other processes, such as the cell cycle, remains to be clarified.

CDKG;2 is the closest homolog of the human CDK11 [5], which was reported to act in splicing [20] but also in other aspects of the cell cycle, such as mitotic spindle formation, centrosome maturation, and bipolar spindle morphogenesis [21]. Moreover, in *Xenopus laevis*, CDK11 regulates the rate of assembly and stabilization of the mitotic spindle through RanGTP, a central protein of the Ran (Ras-related nuclear protein) GTPase network [22].

During cell division, the RanGTPase network governs DNA replication, the mitotic spindle assembly, and the nuclear envelope assembly [23,24,25]. The activity of Ran is determined by cycling between its GTP (guanosine triphosphate) and GDP (guanosine diphosphate)-bound states [26]. Ran intrinsic ability to form RanGDP molecule is very low, being increased by RanGAP (Ran-GTPase–activating protein) [27]. RanBP1 stabilizes RanGTP and enhances RanGAP activity, which is critical for maintaining levels of RanGDP/RanGTP in the cell [28,29]. On the other hand, the formation of RanGTP molecules is catalyzed by the Ran-specific guanine exchange factor (RanGEF) RCC1 (Regulator of chromosome condensation 1) [30].

The correct localization of RanGDP and RanGTP is crucial for the functions regulated by the RanGTPase cycle. The directionality of nucleo-cytoplasmic transport is guaranteed by a higher quantity of RanGTP inside the nucleus and RanGDP in the cytoplasm [30]. When there is no nuclear envelope during mitosis, RanGTP is concentrated only in the vicinity of chromatin to ensure mitotic progression [30]. Therefore, the localization of the Ran system’s proteins is crucial for correctly positioning RanGTP molecules and the downstream events it regulates. Interestingly, the localization of the main proteins in this system is conserved in eukaryotes [31].

Ran-binding protein 1 (RanBP1), a central regulator of the Ran cycle is predominantly cytoplasmic during interphase [26,32,33,34]. Nevertheless, analyses using mutants and nuclear export inhibitors reveal nuclear accumulation of RanBP1, indicating that it shuttles between the nucleus and cytoplasm [26,32,33,34]. Notably, to our knowledge, RanBP1 has not been observed in the nucleus under native conditions (absence of mutations and drugs). During cell division in mammalian cells, RanBP1 is highly concentrated in the mitotic cytoplasm until the early telophase [26]. RanBP1’s localization during cell division in plants has not been reported yet.

Plants with different RanBP1 levels presented reduced size, morphologically abnormal leaves, and altered root growth [35,36,37]. Such phenotypes suggest that RanBP1 also acts on mitosis in plants. In human cells, decreasing RanBP1 levels prevent mitotic entry and cause chromosome segregation errors related to increased microtubule stability and improper localization of cyclin B1 and HURP [38]. Phosphorylation represents a crucial regulatory level for RanBP1. It has been shown that Polo-like kinase-1 (PLK1) phosphorylates RanBP1, and this phosphorylation is crucial for cell division in animals [30,39], as it stabilizes RanBP1 interaction with Ran and allows progression through early mitosis [39]. However, in plants, the regulation of RanBP1 during cell division and its potential phosphorylation by kinases remain largely unexplored.

To contribute to the knowledge about the cell cycle in plants, we focused on characterizing NtCDKG;2 and NtRanBP1 during the cell cycle. Here, we show that NtCDKG;2 localization is regulated during the cell cycle and found in the mitotic spindle during cell division. Additionally, we report its interaction with NtRanBP1 based on yeast two-hybrid (Y2H) and BiFC experiments. We also demonstrate that the localization of NtRanBP1 is regulated during the cell cycle, and, interestingly, we report for the first time that RanBP1 concentrates in the nucleus of plant cells at mitosis onset, exactly when NtRanBP1 and NtCDKG;2 co-localize. These results suggest that NtCDKG;2 may phosphorylate NtRanBP1 at the beginning of mitosis. Together, our data indicate that NtRanBP1 and NtCDKG;2 participate in the regulation of cell division in plants.

## 2. Results

### 2.1. NtCDKG;2 Localization Is Cell Cycle-Related, and During Cell Division, It Localizes to the Mitotic Spindle

In our study of *N. tabacum* flower development, we identified a Cyclin-Dependent Kinase—NtCDKG;2, which interacts with a small protein that regulates cell proliferation—SCI1 [2,5]. The CDKG;2 of Arabidopsis and *N. tabacum* have been associated with the regulation of gene expression through transcription and RNA splicing [5,18,19] but not yet with the control of cell proliferation. NtCDKG;2 is the homolog of the human CDK11 (Supplemental Figure S1) [1]. The human genome has 20 CDKs [40], with two highly similar genes encoding identical CKD11 proteins. Indeed, the phylogenetic tree shows a high-confidence group formed by HsCDK11A, HsCDK11B, and the NtCDKG;2, the focus of the present work (Supplemental Figure S2). In addition to its role in transcription and pre-mRNA splicing, CDK11 regulates the cell cycle [41].

**Figure 1:**
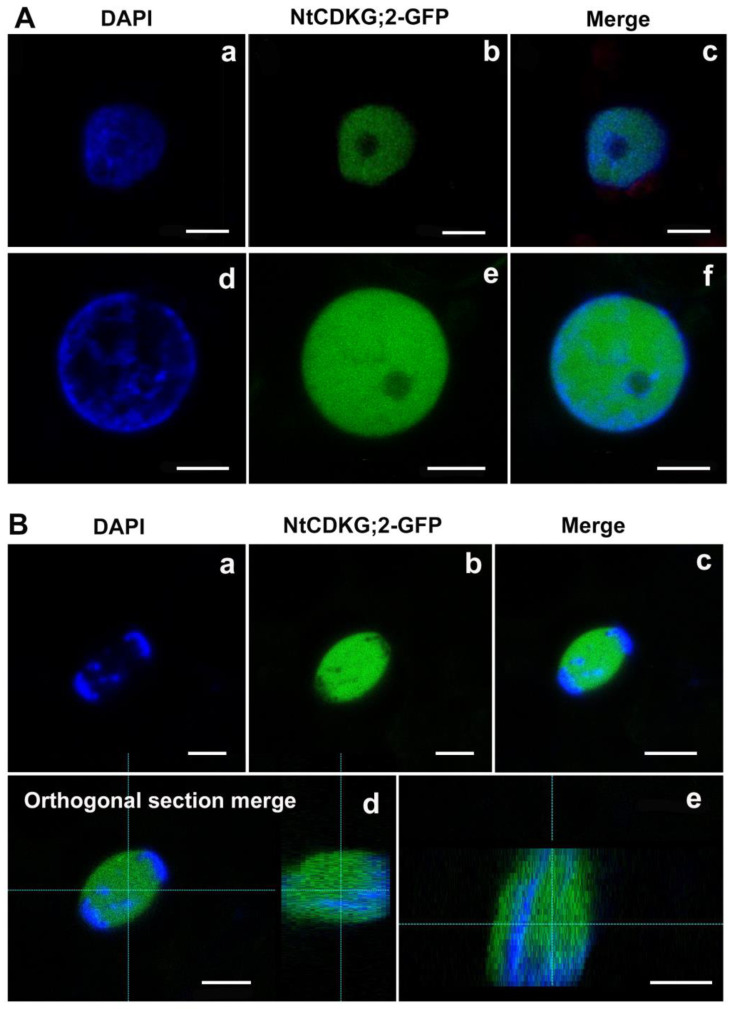
NtCDKG;2-GFP subcellular localization upon transient expression in epidermal cells of *N. benthamiana* leaves. (**A**) At interphase (**a**–**f**), NtCDKG;2-GFP was spread throughout the nucleus. First column: DAPI visualization; second column: visualization of NtCDKG;2-GFP; third column: overlap of the two previous channels. (**B**) At anaphase (**a**–**e**), NtCDKG;2-GFP was localized at the mitotic spindle and adjacent to chromatin. The absence of NtCDKG;2-GFP in the chromatin is demonstrated in detail by an orthogonal cell section (**d**,**e**). (**a**) DAPI visualization; (**b**) visualization of NtCDKG;2-GFP; (**c**) overlap of the two previous channels; (**d**,**e**) orthogonal section on the merge. The images were obtained by confocal microscopy using the Leica TCS SP5 apparatus (Leica Microsystems). Scale bar = 5 µm.

To further elucidate the role of NtCDKG;2 during the cell cycle, localization of NtCDK;2-GFP was first analyzed in transient expression in *Nicotiana benthamiana* leaves (Figure 1). At interphase, the nucleolus is present, and the diffused pattern of DAPI staining indicates decondensed chromatin (Figure 1A). At this stage, NtCDKG;2-GFP was in the nucleus (Figure 1A). Interestingly, during anaphase, NtCDKG;2-GFP localized to the mitotic spindle (Figure 1B) and adjacent to the condensed chromosomes (Figure 1B).

**Figure 2:**
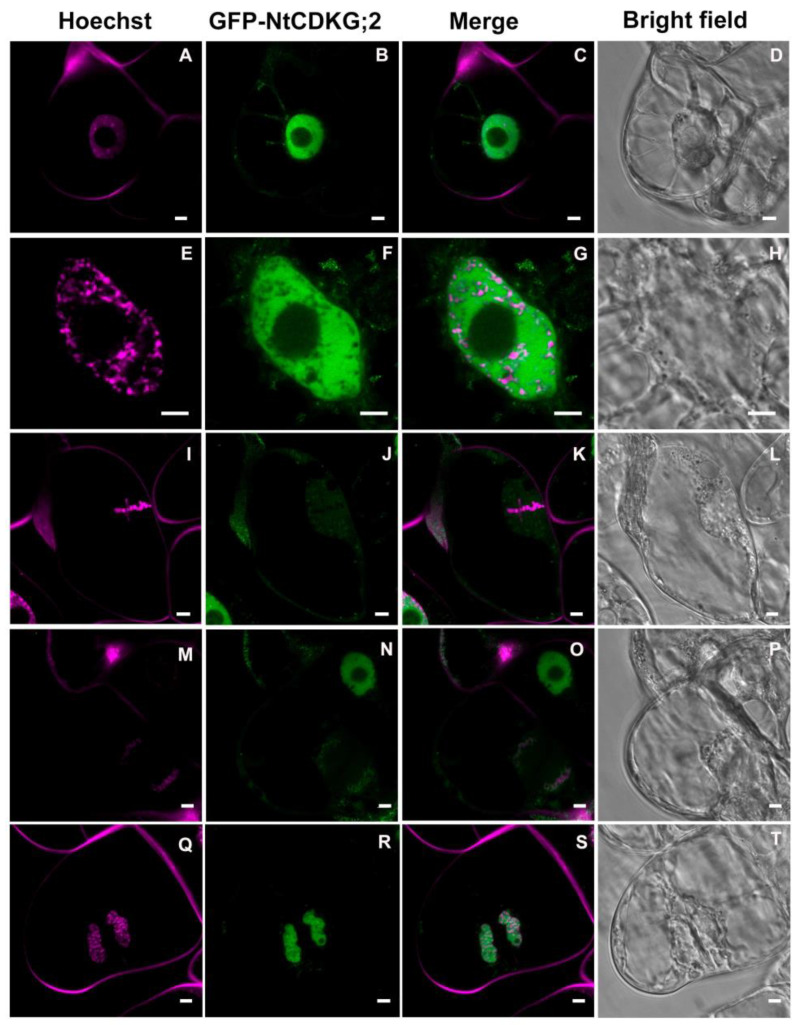
GFP-NtCDKG;2 subcellular localization during the cell cycle in stably transformed BY-2 cells. Chromatin was stained with Hoechst33342. At interphase (**A**–**D**) and early prophase (**E**–**H**), GFP-NtCDKG;2 was spread throughout the nucleus. During metaphase (**I**–**L**), GFP-NtCDKG;2 was absent at the equatorial plane and showed a localization that resembles that of the mitotic spindle. At late anaphase/early telophase (**M**–**P**), GFP-NtCDKG;2 was concentrated close to the chromosomes. In the next phase of the cell cycle (telophase, **Q**–**T**), GFP-NtCDKG;2 was concentrated in the forming nucleus. First column: Hoechst33342 visualization; second column: visualization of GFP-NtCDKG;2; third column: overlap of the two previous channels; fourth column: bright field. The images of BY-2 cells stably transformed with P35S::GFP-NtCDKG;2 were obtained with a Leica TCS SP5 confocal microscope (Leica Microsystems). Bar = 5 µm.

To complement the localization data, we also analyzed BY-2 cells (*N. tabacum* Bright Yellow-2) stably expressing GFP-NtCDKG;2, the inverted fusion configuration, with GFP at the N-terminal of NtCDKG;2. In the interphasic cells, GFP-NtCDKG;2 was found inside the nucleus (Figure 2A–D), as also found in *N. benthamiana* leaves (Figure 1A). At early prophase, chromatin condensation had already started, and GFP-NtCDKG;2 was found mainly in the nucleoplasm (Figure 2E–H). During metaphase, chromosomes were positioned on the equatorial plane, and GFP-NtCDKG;2 was absent at the equatorial plane and localized at the mitotic spindle (Figure 2I–L). At late anaphase/early telophase, when condensed chromosomes were at opposite poles, GFP-NtCDKG;2 was concentrated close to the chromosomes (Figure 2M–P). The nuclear envelope was assembled at the end of mitosis (telophase), and GFP-NtCDKG;2 was concentrated in the nucleus (Figure 2Q–T). Our results show that NtCDKG;2 localization is cell cycle regulated and suggest a role for NtCDKG;2 in mitotic spindle assembly during cell division.

### 2.2. NtCDKG;2 Interacts with NtRanBP1 and Ntβ-Tubulin Proteins

**Figure 3:**
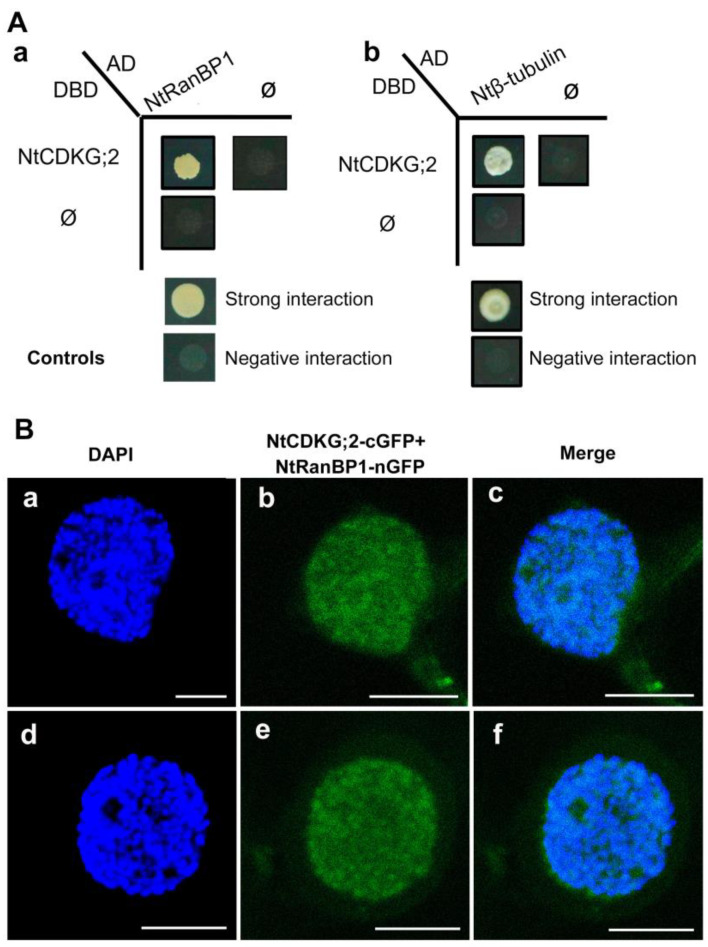
NtCDKG;2 interacts with NtRanBP1 and Ntβ-tubulin. (**A**) Interaction analysis using the Y2H assay. (**a**) Top line: yeast colonies expressing DBD-NtCDKG;2 fusion protein and AD-NtRanBP1 fusion protein or empty AD (Ø—NtCDKG;2 self-activation control). Bottom line: colony expressing AD-NtRanBP1 and containing empty DBD (Ø—NtRanBP1 self-activation control). (**b**) Top line: yeast colonies expressing DBD-NtCDKG;2 fusion protein and AD-Ntβ-tubulin fusion protein or empty DBD (NtCDKG;2 self-activation control). Bottom line: colony expressing AD-Ntβ-tubulin and containing empty DBD (Ntβ-tubulin self-activation control). The interactions were confirmed on the SD medium, which lacked leucine, tryptophan, and histidine. In (**a**), the medium was supplemented with 3-amino1,2,4-triazole (1 mM). Strong positive and negative controls are shown at the bottom of each assay. (**B**) Analysis of the interaction between NtCDKG;2 and NtRanBP1 by BiFC. Epidermal cells of *N. benthamiana* leaves were co-transformed with vectors expressing NtCDKG;2–cGFP and NtRanBP1–nGFP. First column: DAPI visualization. Second column: visualization of the GFP signal due to the interaction of NtCDKG;2–cGFP with NtRanBP1–cGFP. The interaction occurs in the nucleus. Third column: channel overlap. The fluorescence seen outside the nucleus in (**b**,**c**) is due to the autofluorescence of a trichome. The images were obtained with a Leica TCS SP5 confocal microscope (Leica Microsystems). Scale bar = 5 µm.

To contribute to the elucidation of the role of NtCDKG;2, we screened a stigma/style cDNA library in the Y2H system [42], using NtCDKG;2 as bait [5]. Notably, this screening revealed NtRanBP1 and Ntβ-tubulin as potential interaction partners of NtCDKG;2. The interaction of NtCDKG;2 with these proteins was confirmed by a pairwise Y2H test (Figure 3A(a,b)). Moreover, the interaction between NtCDKG;2 and NtRanBP1 was confirmed in planta by BiFC (Figure 3B(a–f)). β-tubulins form the physical structure of microtubules through heterodimers with α-tubulins [43]. RanBP1 is a member of the Ran system, which is highly conserved in eukaryotes and regulates fundamental aspects of the cell, such as nucleocytoplasmic transport of molecules and cell division [24,30,31]. Despite its importance, little has been studied about RanBP1 during the cell cycle in plants, and in *N. tabacum*, no studies have been conducted so far. Moreover, the relationship between RanBP1 and CDKs in plants has not been explored before, leading us to investigate these aspects further.

### 2.3. In Silico Characterization of NtRanBP1

In the *N. tabacum* proteome, we found six RanBP1s sequences corresponding to three groups of genes and their homologs of the *N. tabacum* ancestral genomes, *Nicotiana sylvestris* and *Nicotiana tomentosiformis* (Supplemental Figure S3). They were named RanBP1-1a, RanBP1-1b, RanBP1-2a, RanBP1-2b, RanBP1-3a, and RanBP1-3b (Supplemental Figures S3–S5) based on their similarity to the *N. benthamiana* RanBP1 sequences [44]. The six proteins are encoded by structurally similar genes with four exons and three introns (Supplemental Figure S4). Genes of the same group (homologs of the ancestral genomes, *N. sylvestris*, and *N. tomentosiformis*) have more similarities concerning both sizes of the exons and introns, as well as exon distribution (Supplemental Figure S4). The similarity among the exon sizes leads to proteins with similar sizes (220 to 224 amino acids). The sizes of the UTRs are also similar; only RanBP1-3b has a very small 3’ UTR (Supplemental Figure S4), probably due to incomplete sequencing in the large-scale experiments available at public databases. The main differences are in the last exon that encodes the C-termini, the most distinct part of these proteins (Supplemental Figure S5).

**Figure 4:**
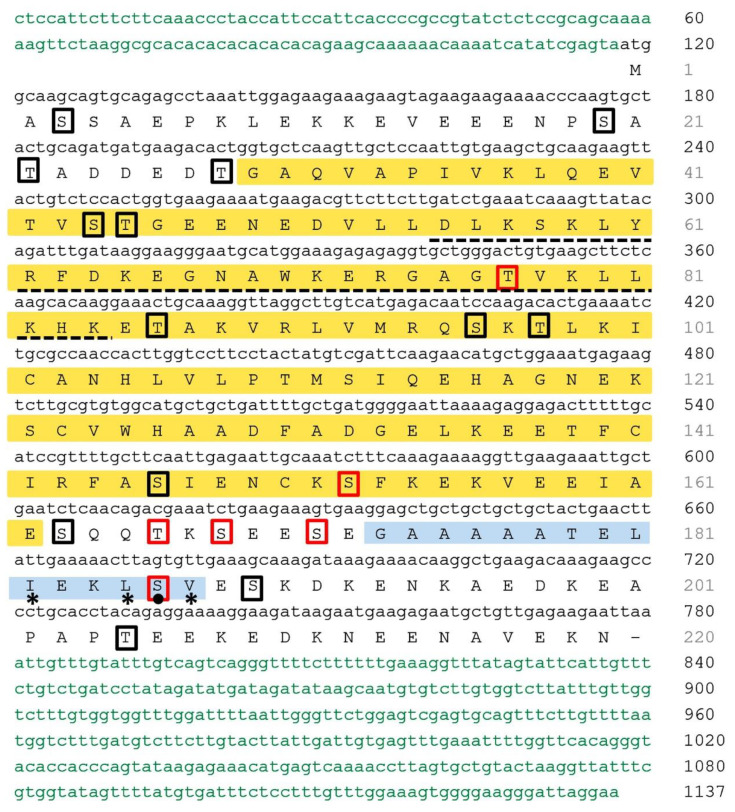
NtRanBP1 cDNA nucleotide and the deduced amino acid sequences showing the functional domains identified by in silico analysis. The coding sequence (CDS) is shown in black, and the untranslated regions (UTRs) are represented in green. NtRanBP1 protein consists of 220 amino acids, with the majority composing the RBD, highlighted in yellow. The black dashed underline indicates the importin α-dependent NLS, identified using the cNLS Mapper software [47]. NtRanBP1 also shows an NES (blue region), identified with the software LocNES [47], and the crucial amino acids for NES functionality in other species are marked by asterisks [33]. Phosphorylation sites are in boxes: black (for a probability of 50%) and red (for a probability of 90%). The identification was done with the software NetPhos 3.1 [48]. The black ball in Serine 186 indicates that this amino acid was found phosphorylated in Arabidopsis RanBP1 [46].

NtRanBP1-1a was identified as the interaction partner of NtCDKG;2 in the Y2H screening. Therefore, the experiments and analyses described in the present work have focused on this gene and its corresponding protein. NtRanBP1-1a encodes a predicted 220 amino acids protein with a molecular weight of 24.4 kDa. In this paper, for the sake of simplicity, NtRanBP1-1a is denominated only as NtRanBP1. Its protein sequence has the RBD between amino acids 29–162 (Figure 4). The region between amino acids 55 and 84, within the RBD, is an importin α-dependent NLS (Figure 4). In addition, NtRanBP1 has an NES with identical hydrophobic residues demonstrated to be functional in AtRanBP1a and crucial for nuclear exclusion [33]. This NES sequence (Figure 4 and Supplemental Figure S5) is C-terminal to its RBD, as in the human RanBP1 sequence, which has been demonstrated to be responsible for its cytoplasmic accumulation [32,45].

Interestingly, this NES sequence is absent in the homologous *Saccharomyces cerevisiae* sequence—YRB1 (Supplemental Figure S6)—and may not be necessary for fungi since the YRB1 nuclear export relies on RBD [32]. Moreover, in the NtRanBP1 protein sequence, there are 19 putative phosphorylated residues with 50% occurrence probability or more, among which six residues have 90% phosphorylation probability (Figure 4) according to NetPhos 3.1 software. Serine at position 186, one of the amino acids strongly predicted to be phosphorylated, is phosphorylated in Arabidopsis [46]. It is important to note that this serine residue is within the NES, which will be discussed below.

### 2.4. NtCDKG;2 and NtRanBP1 Expression During Cell Cycle

**Figure 5:**
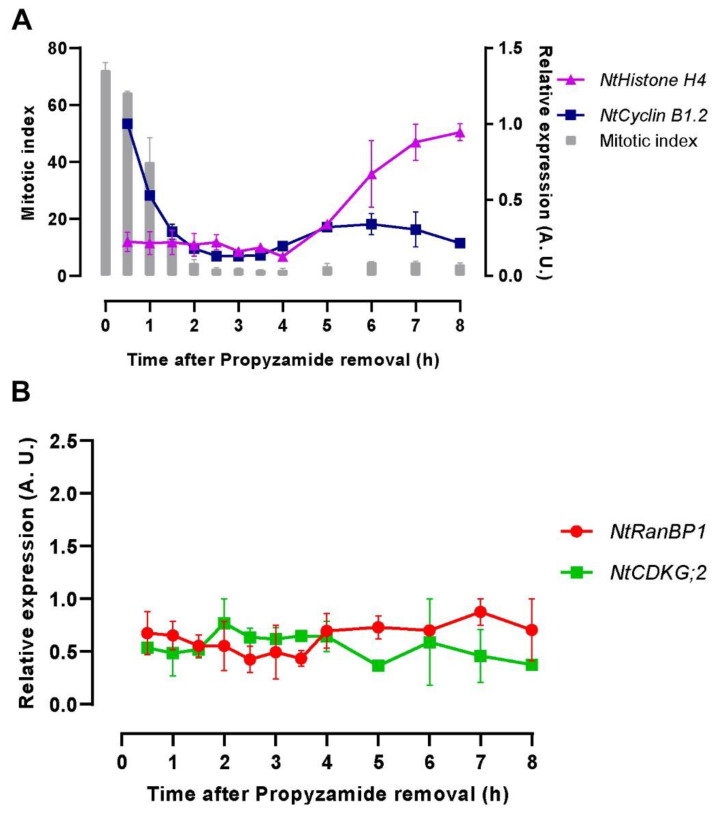
*NtCDKG;2* and *NtRanBP1* expression during the cell cycle. BY-2 cells were synchronized with aphidicolin and propyzamide treatment (for details see Materials and Methods). (**A**) Graphical representation of Mitotic Index and relative expression of the cell cycle markers *NtCyclin B1.2* and *NtHistone H4*. (**B**) Relative expression of *NtCDKG;2* and *NtRanBP1*. The expression of both genes remained stable throughout the cell cycle period analyzed. Values are means ± SEM of biological replicates.

*NtCDKG;2* and *NtRanBP1* expression were analyzed during the cell cycle on synchronized BY-2 cells. As far as we know, this is the first report of plant *RanBP1* expression during the cell cycle. The induction of the synchronization of these cells was done with aphidicolin, which blocks cells at the S-phase, and propyzamide, which stops cells at metaphase [49]. The inhibitory effect of these drugs is lost after removing them from the culture medium; thus, cells can follow to the next phase [49]. The Mitotic Index monitored the synchronization level (Figure 5A). In this analysis, the highest proportion of dividing cells was immediately after propyzamide removal (time 0), when approximately 72% of the cells were in mitosis (Figure 5A). The percentage of cells in division decreased progressively, reaching less than 5% in 2 h after the removal of propyzamide, a value that did not increase until the end of the experiment—8 h after drug withdrawal (Figure 5A). The monitoring of the cell cycle phases was also done by analyzing the expression of the G1/S marker, *NtHistone H4* [50], and the G2/M marker, *NtCyclin B1* [51] (Figure 5A). These analyses demonstrated that the expression of *NtCDKG;2* and *NtRanBP1* genes are not regulated at the mRNA level throughout the cell cycle (Figure 5B).

### 2.5. NtRanBP1 Subcellular Localization Is Cell Cycle-Dependent

**Figure 6:**
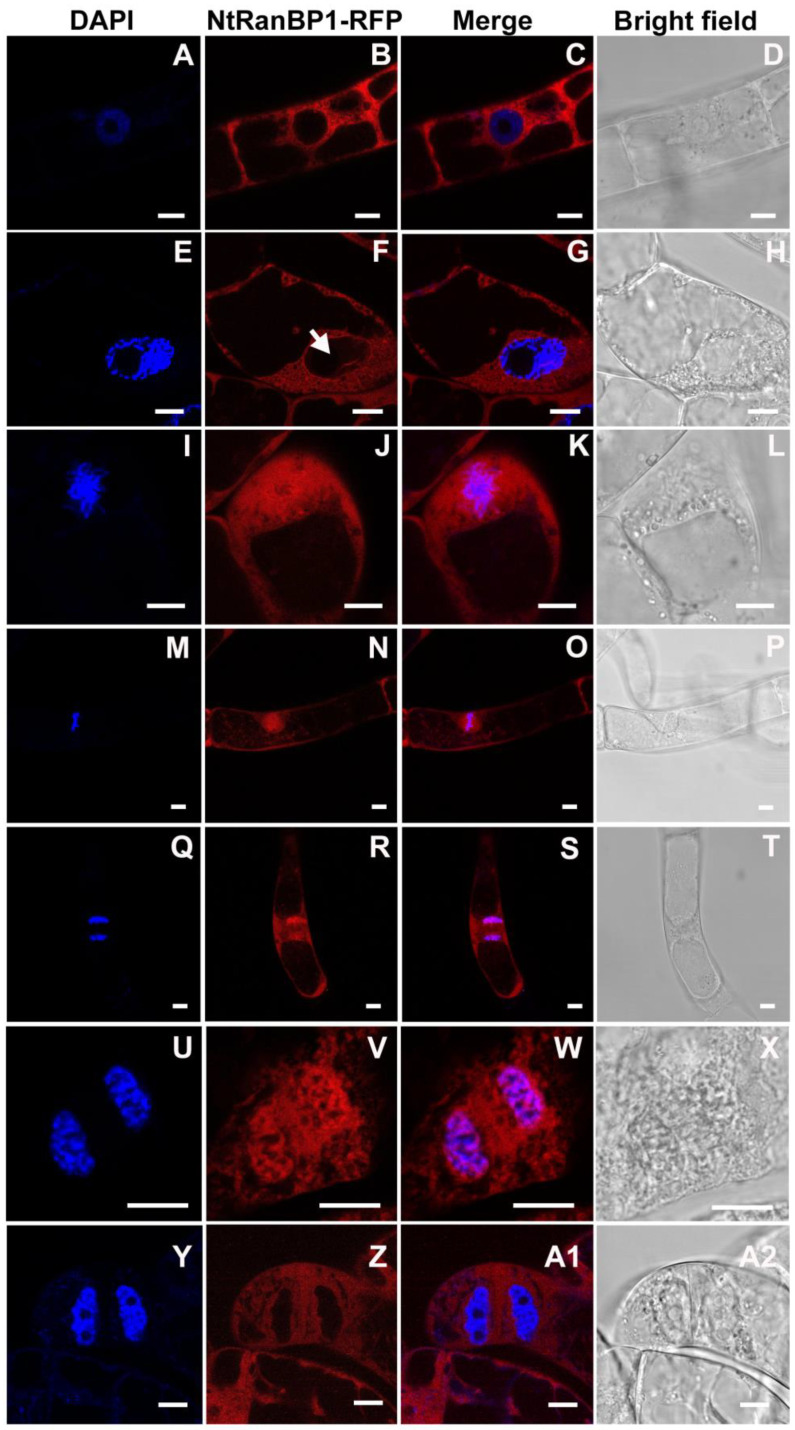
Subcellular localization of NtRanBP1-RFP during the cell cycle in stably transformed BY-2 cells. DAPI was used to stain chromatin. At interphase (**A**–**D**), NtRanBP1-RFP was exclusively cytoplasmic. During prophase (**E**–**H**), NtRanBP1-RFP was found in the cytoplasm and nucleus ((**F**)—arrow). At prometaphase (**I**–**L**), metaphase (**M**–**P**), and anaphase (**Q**–**T**), NtRanBP1 was distributed over the cell, being concentrated in regions close to chromosomes. At telophase (**U**–**X**), NtRanBP1-RFP did not accumulate in one region and was evenly distributed throughout the cytoplasm. In the G1 phase of interphase (**Y**–**A2**), the localization of NtRanBP1-RFP was again exclusively cytoplasmic. First column: DAPI visualization; second column: visualization of NtRanBP1-RFP; third column: overlap of the two previous channels; fourth column: bright field. The images of BY-2 cells stably transformed with P35S::NtRanBP1-RFP were obtained with a Leica TCS SP5 confocal microscope (Leica Microsystems). Bar = 10 µm.

In addition to expression analysis during the cell cycle phases, we evaluated whether the localization of NtRanBP1 is regulated during mitosis. Thus, we produced BY-2 cells stably expressing NtRanBP1-RFP. In Figure 6A–D, the cell nucleus is intact, the nucleolus is evident, and the DAPI-stained chromatin shows a diffuse pattern, which are typical interphase characteristics. At this stage, DNA is not condensed, and NtRanBP1-RFP was found only in the cytoplasm (Figure 6B,C). The nuclear envelope still exists at early prophase, and chromatin starts condensing, with its filaments well distinguished (Figure 6E–H). At this stage, NtRanBP1-RFP was found in the cytoplasm and inside the nucleus, as indicated by the arrow in Figure 6F. During late prophase/prometaphase, the nuclear envelope breaks down, and the condensed chromatin filaments, already seen as chromosomes, migrate to the central area (Figure 6I–L). At the same time, NtRanBP1-RFP accumulates in regions adjacent to chromosomes (Figure 6J–K). At metaphase, the chromosomes are aligned in the equatorial plane of the cell, and NtRanBP1-RFP is concentrated in the regions adjacent to the chromosomes (Figure 6M–P). Although there is a high concentration of NtRanBP1-RFP near chromatin, few molecules co-localize with it (Supplemental Figure S7). At anaphase, the condensed chromosomes are at opposite poles, and NtRanBP1-RFP remains accumulated in the regions adjacent to the chromosomes (Figure 6Q–T). At telophase, the nuclear envelope reassembles around each set of chromosomes, separating the nuclear DNA from the cytoplasm; the cell chromatin is still dense, and the nucleolus is absent. At this stage, NtRanBP1-RFP was evenly distributed throughout the cytoplasm (Figure 6U–X). Subsequently, the cell has a fully formed nuclear envelope, and the nucleoli are clearly visible (Figure 6Y–A2), which are the characteristics of G1. At this point of the cell cycle, NtRanBP1-RFP was found exclusively in the cytoplasm (Figure 6Z,A1).

### 2.6. NtCDKG;2 and NtRanBP1 Co-Localize in the Nucleus at Mitosis Onset

**Figure 7:**
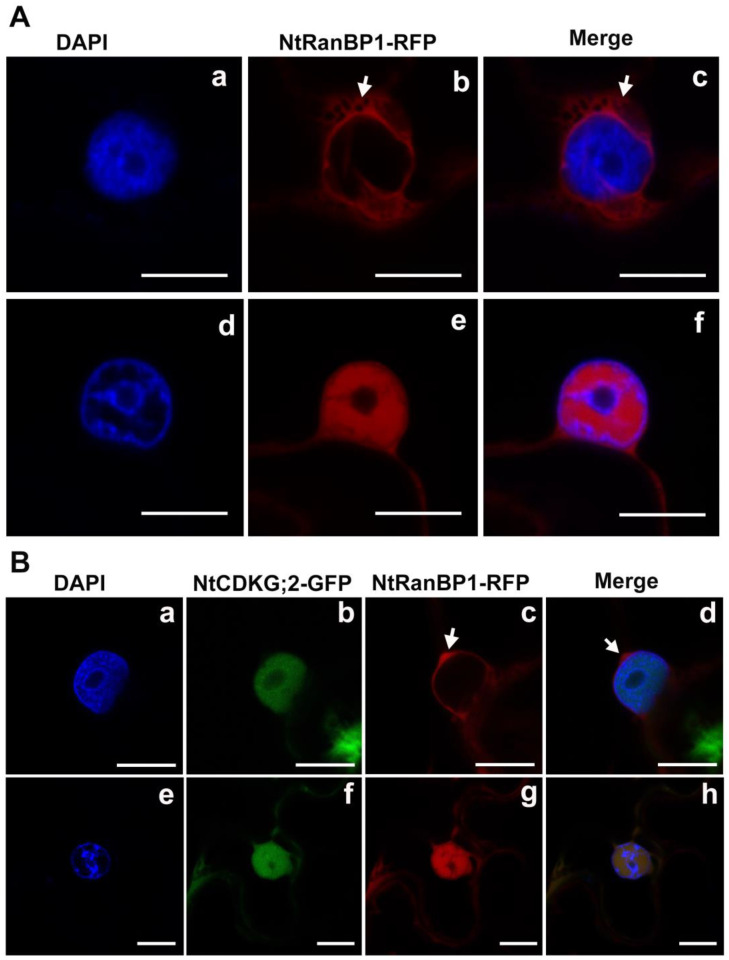
Subcellular localization of NtRanBP1-RFP in transiently transformed epidermal cells of *N. benthamiana* leaves. (**A**) NtRanBP1-RFP localizes exclusively in the cytoplasm during interphase (**a**–**c**), but accumulates in the nucleus at the onset of cell division, marked by chromatin condensation (**d**–**f**). First column: DAPI visualization; second column: visualization of NtRanBP1-RFP; third column: channel overlap. (**B**) Co-localization assay of the proteins NtCDKG;2-GFP and NtRanBP1-RFP. Their co-localization is cell cycle-dependent, occurring specifically at the onset of mitosis (**e**–**h**). NtCDKG;2-GFP is always nuclear (**b**,**f**), whereas NtRanBP1-RFP is only present in the nucleus during chromosome condensation (**e**,**g**). At interphase, NtRanBP1-RFP is found in the cytoplasm ((**c**,**d**)—arrow). First column: DAPI visualization; second column: visualization of NtCDKG;2-GFP; third column: visualization of NtRanBP1-RFP; fourth column: channel overlap. The images were obtained by confocal microscopy using the Leica TCS SP5 apparatus (Leica Microsystems). Scale bar = 10 µm.

To investigate the localization of RanBP1-RFP in the nucleus even further (Figure 6F), we decided to study its localization by transient expression in epidermal cells of *N. benthamiana* leaves. These cells have a longer cell cycle, and the expression level is higher than in BY-2 stably transformed to express RanBP1-RFP. The interphase and mitosis phases were defined using the DAPI-stained chromatin distribution. During interphase, in which DAPI is diffuse and evenly distributed (Figure 7A(a–c)), NtRanBP1-RFP is exclusively cytoplasmic (Figure 7A(b,c)). In the cell shown in Figure 7A(d–f), DAPI is concentrated in some nuclear regions, indicating chromatin condensation and the beginning of cell division. At this point in the cell cycle, NtRanBP1-RFP is clearly localized within the nucleus (Figure 7A(e,f)), establishing that NtRanBP1-RFP accumulates in the nucleus at the onset of cell division.

To interact, as revealed by the Y2H screening, NtCDKG;2 and NtRanBP1 must be in the same cellular compartment. To capture the stage at which they co-localize, we performed the transient expression of NtCDKG2-GFP and NtRanBP1-RFP in *N. benthamiana* leaf epidermal cells. Our analysis revealed the same localization patterns observed in individual assays (Figure 1 and Figure 7A). At interphase, NtCDKG;2-GFP is in the nucleoplasm, and NtRanBP1-RFP is in the cytoplasm (Figure 7B(a–d)). During early mitosis, when chromatin begins to condense, NtRanBP1-RFP is found in the nucleus, along with NtCDKG;2-GFP (Figure 7B(e–h)). These findings indicate that NtCDKG;2 and NtRanBP1 may interact exclusively during cell division.

## 3. Discussion

### 3.1. The Emerging Role of NtCDKG;2 in Mitotic Spindle Regulation

In recent years, some studies have shown the role of CDKG;2 in RNA splicing [5,16,18,19], but the involvement of this protein in other cellular processes has not been described. During the cell cycle, NtCDKG;2 localization changes from nuclear distribution at interphase to concentrated in the mitotic spindle region at metaphase and anaphase (Figure 1 and Figure 2). In these stages of cell division, NtCDKG;2 is positioned adjacent to the chromosomes (Figure 1). NtCDKG;2 localization during cell division and the identification of Ntβ-tubulin and NtRanBP1 as its interaction partners (Figure 3) point to a role in the mitotic spindle.

NtCDKG;2 is the plant homolog of the human CDK11 (Supplemental Figures S1 and S2). Remarkably, CDK11 functions in diverse roles, such as coordinating transcription and mRNA processing, as well as regulating mitosis and apoptosis [21,22,52,53,54]. CDK11 is required for the transcription of histone genes during the S phase, and its absence results in the accumulation of cells in G1 [55,56]. Intriguingly, CDK11 is required for gametogenesis and fertility in *Caenorhabditis elegans* [54]. In *X. laevis*, CDK11 stabilizes microtubules during cell division, and, in its depletion, the spindle assembly rate is reduced [22]. It is important to highlight that CDK11’s action on microtubule stabilization depends on RanGTP, whose production and regulation are done by the Ran system [22]. In this system, RanBP1 functions as one of the essential regulatory proteins [24]. Here, we demonstrate that the similarities between CDK11 and NtCDKG;2 are not restricted to the primary amino acid sequence but extend to the mitotic spindle localization and the interaction with the Ran system. Therefore, CDK11 and its plant homolog CDKG;2 are fascinating versatile CDKs with multiple roles, including mitotic spindle regulation. Additional experiments will be necessary to confirm the role we propose for NtCDKG;2 in mitotic spindle regulation.

### 3.2. NtRanBP1 Spatiotemporal Regulation and Nuclear Arrest at Mitosis Onset

The Ran/RanBP1/RanGAP1 signaling network is a central regulator of nucleocytoplasmic transport of proteins and RNAs during interphase [57,58]. The Ran network components also regulate the timing of cell cycle transitions and, in mitosis, are essential to assembling the mitotic spindle and nuclear-envelope dynamics [23,24,26]. It has already been shown that mitotic progression requires the precise positioning of components and effectors of the Ran network at specific sites of the mitotic apparatus [24,57]. RanBP1 is a crucial component of this signaling network and contributes to establishing the RanGTP gradient and appropriate localization of mitotic regulatory factors on spindle microtubules [38]. It is known that RanBP1 regulates mitotic spindle assembly, controls microtubule dynamics during metaphase and anaphase, and facilitates nuclear chromatin reorganization after mitosis [59].

The expression of *NtRanBP1* is not transcriptionally regulated during the cell cycle in plant cells, at least within the time frame analyzed (Figure 5), as in mammalian cells in which the mRNA level peaks at the S phase and decreases during mitosis [60,61]. However, RanBP1 subcellular localization is clearly cell cycle-related in plant cells (Figure 6 and Figure 7). It is assumed that RanBP1 shuttles continuously between the cytoplasm and nucleus, as demonstrated in yeast [32]. However, images of RanBP1 on the nucleus were only reported using mutants or drugs [26,32,33]. To our knowledge, our results represent the first time that RanBP1 was documented in the nucleus of plant cells without any drug or mutation of its endogenous sequence in other organisms. The fact that we are studying the expression of a fusion protein (NtRanBP1-RFP) under the control of a strong promoter (CaMV 35S) seems not to have affected the RanBP1 localization in the cytoplasm during interphase, which is consistent with previous reports in the literature. This result suggests that the NtRanBP1 overexpression did not overload the nuclear export machinery. It also implies that the fusion with RFP has not concealed important signals necessary for correct subcellular RanBP1 localization. Therefore, we assume that the localization observed in our experiments for NtRanBP1-RFP truly represents the localization of the endogenous RanBP1 protein. Future in planta experiments, such as the elegant analysis of the cell cycle in root apical meristem cells [62], could provide further insights into the localization dynamics of NtRanBP1 throughout the cell cycle.

Additionally, the nuclear localization of RanBP1 reported here is associated with a specific stage of cell division. At early mitosis, NtRanBP1-RFP evidently concentrates inside the nucleus, behind the still present nuclear envelope, outside the chromatin, and in its vicinity (Figure 7 and Supplemental Figure S7). This localization must be essential to ensure the proper RanGTP distribution, which is necessary to release the spindle assembly factors. Therefore, the concentration of RanBP1 inside the nucleus must be crucial for correct spindle assembly and mitosis progress. We propose that, at the beginning of cell division, some signaling modifies the intracellular condition and/or directly modifies the proteins so that RanBP1 is no longer efficiently transported to the cytoplasm and is subsequently arrested inside the cellular nucleus. What are the factors regulating RanBP1 transport to the cytoplasm?

The RanBP1 is a small protein that could passively diffuse into the nucleus. Still, it has already been demonstrated that it enters the nucleus by active transport, driven by the NLS present at the RBD (reviewed in [59]). RanBP1 release from the nucleus is conditioned by the NES in animals, as well as in plants. Ref. [34] demonstrated that the C-terminus of the *A. thaliana* AtRanBP1c, which contains the NES, is required for its cytoplasmic localization. Interestingly, the AtRanBP1c was shown to be critically involved in regulating mitotic progression [35]. The conserved animal and plant NES sequence is also present in NtRanBP1 (Supplemental Figure S6) and should be responsible for its cytoplasmic localization during interphase. Therefore, what is the nature of the modification occurring at the onset of mitosis that results in NtRanBP1 nuclear arrest?

### 3.3. The Role of Phosphorylation in Regulating NtRanBP1 and Its Potential Targeting by NtCDKG;2

In human cells, phosphorylation of RanBP1 by Polo-like kinase-1 (PLK1) stabilizes the interaction with Ran and is required for early mitotic progression [39]. RanBP1 phosphorylation by PLK1 occurs in a mitosis-specific manner and is required for proper microtubule formation during early mitosis [39]. Additionally, it was demonstrated in *X. laevis* that RanBP1 forms a stable complex with Ran and RCC1 (the RRR complex) during mitosis [30]. Phosphorylation of XlRanBP1 at the metaphase–anaphase transition releases RanBP1 from the RRR complex, allowing RCC1 to bind to the chromatin to form RanGTP [30,63]. At the end of mitosis, RanGTP is involved in the assembly of the nuclear envelope [30,63]. In addition, RCC1 and RanGAP, other Ran regulatory proteins, are phosphorylated by CDK during mitosis in humans [64,65]. Hence, phosphorylation is an important mechanism regulating the Ran network. We propose that the arrest of NtRanBP1 on the nucleus (Figure 7) is signaled by phosphorylation during the onset of mitosis. However, plants do not have a PLK1 homolog [66], and the specific serine/threonine kinase that may phosphorylate RanBP1 in plants is still an open question.

**Figure 8:**
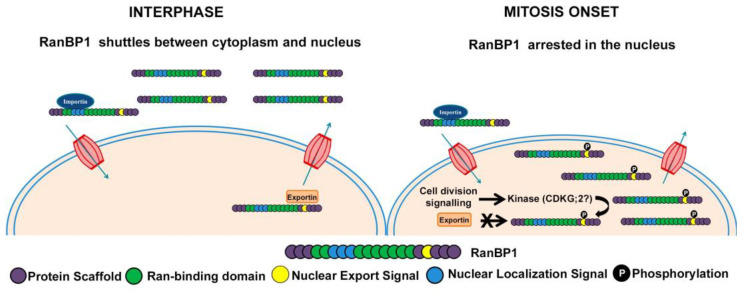
The proposed model for RanBP1 localization depends on the cell-cycle phase. During interphase, NtRanBP1 dynamically shuttles between the cytoplasm and nucleus. This regulatory mechanism is mediated through NLS and NES. At the onset of mitosis, we hypothesize that phosphorylation events—potentially performed by NtCDKG;2—inhibit exportin-mediated transport, resulting in the retention of NtRanBP1 within the nucleus. This precise regulation of NtRanBP1 localization during cell division, as observed in other organisms, would be critical for ensuring proper cell cycle progression.

As shown in Supplemental Figure S6, NtRanBP1 has an NES similar to the NES present in the human RanBP1. It has identical amino acids at crucial positions, which have been demonstrated to be active [33]. During interphase, NtRanBP1 is cytoplasmic (Figure 7A(b),B(c)). Therefore, we assume that its NLS (Supplemental Figures S5 and S6) is not functional or, most likely, functional, but the NES is prevalent during this cell cycle phase. At the beginning of cell division, while the nuclear envelope is still present and chromatin is being condensed (early prophase), NtRanBP1 becomes clearly concentrated in the nucleus (Figure 7A(e),B(g)), suggesting that the signaling for NtRanBP1 shuttling is interrupted at this point. Both NLS and NES have a residue with a high probability of being phosphorylated, T77 and S186, respectively (Figure 4). Interestingly, the residue S186, located at the NES of Arabidopsis and rice RanBP1, was demonstrated to be phosphorylated [46]. Considering that NtCDKG;2 is an interaction partner of NtRanBP1 (Figure 3), we propose that a cell division signal promotes the phosphorylation of NtRanBP1 by NtCDKG;2, probably at the NES, which results in NtRanBP1 nuclear arrest (Figure 8). This hypothesis opens promising directions for experimental validation to uncover the regulatory mechanisms governing the NtCDKG;2 and NtRanBP1 interaction. In particular, the functional validation of the putative NLS and NES of NtRanBP1 and the proposed phosphorylation at the NES remains to be established. For this, targeted mutagenesis of phosphorylation sites (e.g., Ser186) and the NLS of NtRanBP1 can be key in deciphering the regulatory mechanisms underlying its localization and function. Evaluating the kinase activity of NtCDKG;2 on NtRanBP1 will also be crucial for understanding the molecular regulation mediated by this putative cyclin-dependent kinase. Moreover, analysis using native promoter-driven expression and CRISPR-edited lines of NtCDKG;2 and NtRanBP1 would provide more insights into the endogenous behavior of these proteins and disruptions in the cell cycle.

In summary, this study elucidates the dynamic localization of NtCDKG;2 and NtRanBP1 throughout the cell cycle, highlighting the nuclear retention of NtRanBP1 at mitosis onset. Furthermore, the interaction between NtCDKG;2 and NtRanBP1 suggests a phosphorylation-dependent nuclear retention of NtRanBP1 mediated by NtCDKG;2. This work also proposes a potential regulatory role for NtCDKG;2 in mitotic spindle assembly contributing to cell cycle progression in plants. These findings are consistent with prior studies on the functional impact of altered expression of *CDKG;2* and *RanBP1* in plants. Silencing of *RanBP1* causes growth retardation and abnormal leaf morphology in *N. benthamiana* [36] and mitotic arrest in the roots of *A. thaliana* [35], while its overexpression in *Populus deltoides* leads to growth retardation due to an increased proportion of cells in the G2 phase [37]. Moreover, *CDKG;2* mutants in *A. thaliana* exhibit delayed organogenesis, failure to form roots, and defective reproductive structures [17]. In conclusion, our study reveals a novel interaction between NtCDKG;2 and NtRanBP1, along with their detailed localization throughout the cell cycle. Our findings set a path forward to better understanding the roles of these proteins in the plant cell cycle, a vital process for plant development and growth.

## 4. Materials and Methods

### 4.1. Plant Materials and Growth Conditions

*N. benthamiana* was used for protein localization and co-localization experiments. Seeds were sown in Bioplant^®^ substrate with vermiculite, watered in trays from below, and cultivated in a Weiss Gallenkamp growth chamber under the following conditions: 16 h light/8 h dark photoperiod, at 500 μmol m^−2^ s^−1^ light, 22 °C temperature, and 55% humidity. Individual plants were used for the experiments 70–90 days after sowing.

*N. tabacum* cv. Bright Yellow-2 (BY-2) cells were used in stable transformation and cell cycle synchronization protocols. This suspension culture was maintained in a liquid medium optimized for BY-2 cells, essentially as described by [67]: MS plant salt mixture (0.5 g/L), 87.6 mM sucrose, 1.32 mM KH_2_PO_4_, 3 µM thiamine HCl, 0.55 mM myo-inositol, 0.01 mM 2,4-dichlorophenoxyacetic acid (2,4-D), and 2.56 mM MES. Cultures were maintained at 28 °C, shaking at 150 rpm in darkness, and subcultured every seven days.

### 4.2. Plasmid Constructs

The gene constructions used in the present work were prepared in plant Gateway vectors provided by [68] or yeast Gateway vectors from Thermo Fisher Scientific, Waltham, MA, USA. The molecular biology techniques were performed as described in [69]. All gene constructions were sequenced to confirm their integrity, in-frame gene fusions, and the absence of undesired mutations.

### 4.3. Transient Expression in N. benthamiana Leaves

Transient expression assays in *N. benthamiana* leaves were conducted to investigate the localization of NtRanBP1-RFP and NtCDKG;2-GFP and their co-localization. Transient expression assays were also performed to analyze the interaction between NtRanBP1 and NtCDKG;2 using BiFC experiments. *Agrobacterium tumefaciens* pGV2260 cells carrying the constructs P35S::NtRanBP1-RFP and P35S::NtCDKG;2-GFP for localization and co-localization assays, or P35S::NtCDKG;2-cGFP and P35S::NtRanBP1-nGFP for BiFC analysis were used separately or in combination for leaf infiltration, as described by [70]. Following infiltration, the plants were maintained at room temperature and under light conditions for 48 to 72 h, after which the leaves were treated with 4ʹ,6-diamidino-2-phenylindole (DAPI). Confocal microscopy analysis was performed using a Leica TCS SP5 microscope (Leica Microsystems, Wetzlar, Germany, LMMC—FMRP/USP). DAPI, GFP, and RFP signals were detected using emission spectra of 425–450 nm, 500–550 nm, and 630–680 nm, respectively. Localization, co-localization, and BiFC experiments were performed in at least three independent biological replicates, with the figures representing consistent results from these replicates.

### 4.4. Stable Transformation of BY-2 Cells

BY-2 cells were stably transformed with P35S::GFP-NtCDKG;2 and P35S::NtRanBP1-RFP constructions. For the transformation, BY-2 cultures in exponential growth were incubated for 48 h in the dark at room temperature with 100 µM acetosyringone, and *A. tumefaciens* pGV2260 transformed with the selected constructions. After incubation, cells were washed and plated on MS solid medium supplemented with kanamycin (50 µg/mL) in combination with cefotaxime (100 μg/mL) and incubated in the dark at room temperature. *Calli* formed after one month were subsequently transferred to fresh antibiotic-supplemented plates. Selected *calli* were cultured in a liquid medium for several subcultures to establish stable transgenic cell lines. The transformed cells were treated with 4ʹ,6-diamidino-2-phenylindole (DAPI) and analyzed for protein localization experiments. Fluorescence was analyzed using a Leica TCS SP5 confocal microscope (Leica Microsystems, LMMC-FMRP/USP). DAPI, GFP, and RFP signals were detected, as mentioned above. Each experiment was conducted with a minimum of three independent biological replicates, with the figures representing consistent results from these replicates.

### 4.5. BY-2 Cell Culture Synchronization

BY-2 cell cultures were synchronized using the two-step method: first with aphidicolin, a DNA polymerase α inhibitor, and then with propyzamide, which inhibits microtubule assembly. The synchronization experiment was conducted in duplicate. The protocol used was adapted from [49,71]. Stock solutions of aphidicolin (10 mg/mL) and propyzamide (1.54 mg/mL) were prepared in dimethyl sulfoxide (DMSO) and stored at 4 °C until use.

For the experiment, 10 mL of stationary-phase BY-2 culture was diluted into 95 mL of MS medium. Aphidicolin was added to a final concentration of 5 µg/mL, and the culture was incubated at 25 °C with shaking at 130 rpm for 24 h. Afterward, aphidicolin was removed by sequential washes with MS medium, and the culture was diluted back to 10 mL in 95 mL of fresh medium for a 5 h incubation. Propyzamide was added to a final concentration of 770 ng/mL, and the culture was incubated for an additional 4 h. Following propyzamide removal by washing, the culture was again diluted into 95 mL of MS medium and maintained at 25 °C with shaking until the experiment’s conclusion.

Sampling was conducted at 30 min intervals during the first 4 h and every hour after that. Aliquots (three replicates) were collected for gene expression analysis and monitoring of the mitotic index (MI) at each time point. The zero-time point was defined as the moment immediately after propyzamide removal and the resuspension of cells in a fresh MS medium.

Cell cycle progression was monitored using the MI. For MI analysis, cells were fixed with Carnoy’s fixative (7.5 mL ethanol and 2.5 mL acetic acid) and stored at 4 °C. DNA staining was performed with 0.1% or 0.2% lacto-propionic-orcein. For MI calculation (number of cells in mitosis/total cells), at least 500 cells were counted per sample. MI analysis was conducted in technical triplicates, and the values were averaged. Additionally, cell cycle progression was assessed by monitoring the expression of *NtHistone H4*, a G1/S phase marker [50], and *NtCyclin B1*, a G2/M phase marker [51].

### 4.6. Y2H Screening and Binary Assays with the Interaction Candidates

The screening of the *N. tabacum* stigma/style cDNA library in the Y2H system was performed as previously described [42], using NtCDKG;2 in the pDEST32 vector (Thermo Fisher Scientific) as bait. The positive clones were Sanger-sequenced with vector-specific primers. The obtained sequences were analyzed using TBLASTX searches against the National Center for Biotechnology Information (NCBI) database (http://www.ncbi.nlm.nih.gov/ (accessed on 21 September 2018) with default parameters to identify the candidate interactions.

Binary (pairwise) Y2H assays were conducted essentially as described by [72] to confirm the interactions revealed during screening (NtRanBP1 and Ntβ-tubulin). Yeast cells of the PJ69-4α strain [73], containing the LacZ and HIS3 reporter genes, were transformed with the following: pDEST32-NtCDKG;2 (in fusion with GAL4 DNA binding domain—DBD), and pDEST22-NtRanBP1 (in fusion with GAL4 DNA activation domain—AD); pDEST32-NtCDKG;2, and pDEST22-Ntβ-tubulin; and different combinations of their empty vectors, as self-activation controls (to test for false positive results). Three independent yeast colonies of each transformation were cultured in a liquid medium and spotted in a selective medium (without leucine, tryptophan, and histidine) at the same concentration (OD_600nm_ = 0.2). The plates were incubated at 30 °C for 2–3 days and observed daily to follow their growth. Additionally, 3-amino-1,2,4-triazole (3AT), a histidine biosynthesis inhibitor, was used to minimize self-activation when necessary.

Interaction controls from the ProQuest™ Two-Hybrid System kit (Thermo Fisher Scientific) were included, featuring the mouse protein Krev1 with DBD and RalGDS variants linked to AD: the wild-type as a positive control (strong interaction) and the M2 variant as a negative control.

### 4.7. In Silico Analysis of NtRanBP1 Sequences

The full-length coding DNA sequence (CDS) of NtRanBP1 cloned in our laboratory (gene accession number LOC107809165) was used as a query in MEGABLAST searches to identify highly similar sequences in *N. tomentosiformis*, *N. sylvestris*, *N. benthamiana*, and *A. thaliana* genomes available at the NCBI database.

The putative NtRanBP1 protein sequence was predicted from the mRNA sequence using the ExPASy Translate Tool (https://web.expasy.org/translate/ (accessed on 21 September 2018). The resulting amino acid sequence was analyzed using LocNES v1.0 [47] (http://prodata.swmed.edu/LocNES/LocNES.php (accessed on 21 September 2018) to identify classical NES with a cutoff score of 0.1, the NetPhos 3.1 Server [48] (https://services.healthtech.dtu.dk/services/NetPhos-3.1/ (accessed on 21 September 2018) to predict phosphorylation sites on serine, threonine, and tyrosine residues with a cutoff score of 0.5, and the cNLS Mapper [74] (https://nls-mapper.iab.keio.ac.jp/cgi-bin/NLS_Mapper_form.cgi (accessed on 21 September 2018) to identify importin α-dependent nuclear localization signals (NLS) with a cutoff score of 6. The Ran-binding domain (RBD) was identified with the CD-Search Tool from NCBI’s Conserved Domain Database (CDD).

Genomic sequences of *N. tabacum* RanBP1 genes were retrieved from NCBI (accession numbers LOC107809165, LOC107771336, LOC107803309, LOC107813247, LOC107814775, LOC107831968), including information on exon and intron positions and untranslated regions (UTRs). Sequence alignments were performed using Clustal Omega v1.2.4 (http://www.clustal.org/omega/ (accessed on 23 December 2024)) with default parameters, and the results were formatted for visualization using BoxShade (https://embnet.vital-it.ch/software/BOX_form.html (accessed on 28 March 2019).

### 4.8. Phylogenetic Analysis

To identify RanBP-containing sequences, we used the consensus Ran-binding domain sequence from the NCBI CDD (cd13179) as a query in BLASTP (BLAST 2.12.0+) searches against the *A. thaliana* Araport11 database, the *N. tabacum* TN90 genome database at NCBI (GCF_000715135.1), the *N. benthamiana* database at the Nicomics webserver [75], the *N. sylvestris* and *N. tomentosiformis* proteomes published by [76], the human (*H. sapiens*) and mouse (*Mus musculus*) canonical proteomes at UniProt (UP000005640 and UP000000589, respectively), and the *X. laevis* genome database at NCBI (GCF_017654675.1). All parameters used were default except for the e-value cutoff, set at 0.00001. Matching sequences were filtered by selecting unique IDs and discarding those larger than 25% of the largest known RanBP1 (228 residues) and smaller than 25% of the smallest (201 residues). The remaining accessions’ corresponding amino acid sequences were retrieved using BLASTDBCMD (BLAST 2.12.0+), and the complete accession table is available in Supplemental File S1.

Human CDK amino acid sequences were obtained from NCBI using accession numbers listed in [40]. The NtCDKG;2 was retrieved from NCBI (accession number XP_016508954.1, gene LOC107826481).

For both RanBP and CDK sequences, global alignments were performed using MAFFT v7.505 [77] with 10,000 refinement iterations. The aligned sequences were then used to construct maximum-likelihood trees with IQ-TREE 2.2.2.7 [78], employing automatic model selection (-m MFP). Branch support was assessed using 1000 ultrafast bootstrap replicates (UFBoot2 [79]) and 1000 replicates of the approximate likelihood-ratio test (aLRT [80]) to provide complementary statistical evaluations of the phylogenetic trees’ reliability. Branches with support values exceeding 80% from both methods were considered statistically significant, a widely accepted cutoff of statistical solid support in phylogenetic studies [78]. The phylogenetic trees were visualized using iTOL v6 [81]. To enhance clarity, non-RanBP1 sequences were pruned from the final RanBP tree.

## Data Availability

The data and materials supporting this study’s findings are available on request from the corresponding author (MHSG).

## Supplementary Materials

The following supporting information can be downloaded at: https://www.mdpi.com/article/10.3390/ijms26010046/s1.
