# Supplementary material for: Unveiling the Movement of RanBP1 During the Cell Cycle and Its Interaction with a Cyclin-Dependent Kinase (CDK) in Plants
Source: Int J Mol Sci. 2024 Dec 24;26(1):46. doi: 10.3390/ijms26010046 (PMC11720235; doi:10.3390/ijms26010046)
Supplement: Supplementary file 1 [file ijms-26-00046-s001.zip › Supplemental Figures - Int J Mol Sci.pdf]

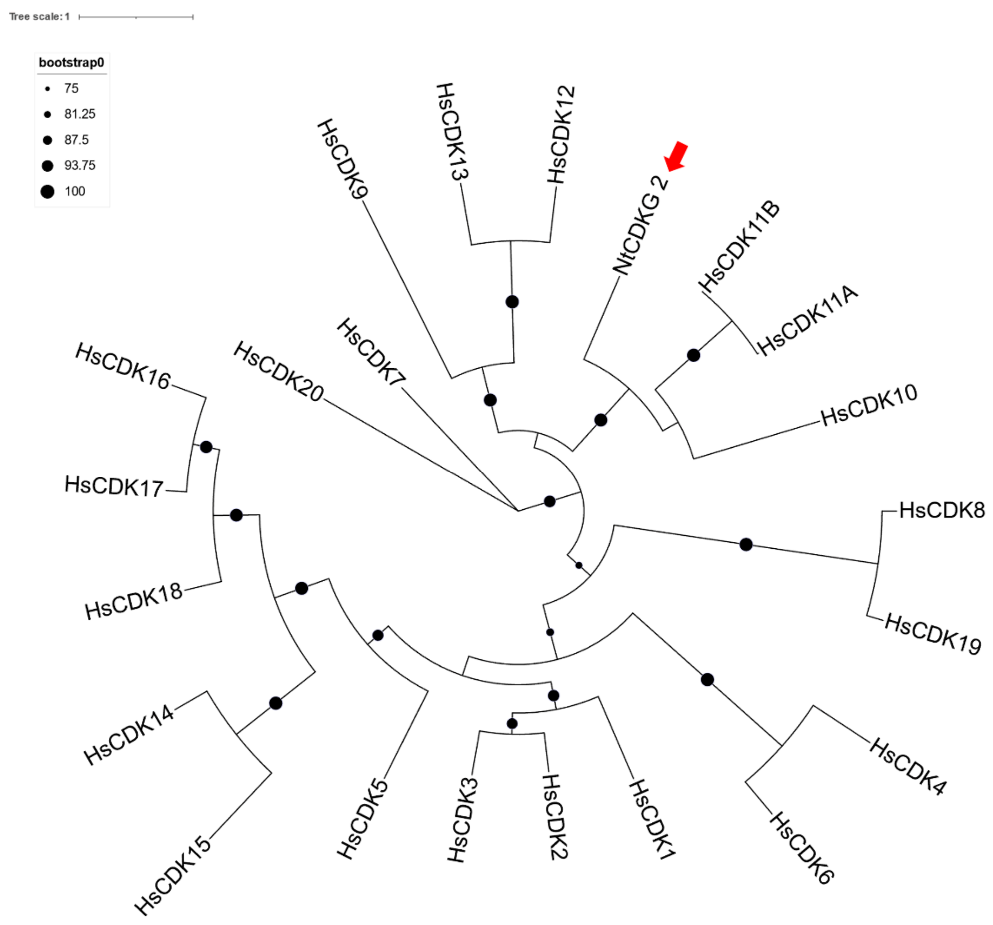

Supplemental Figure S1: Maximum-likelihood tree of CDK sequences from *H. sapiens* and the *N. tabacum* NtCDKG;2 focus of this study (red arrow). Bootstrap values are represented as black circles of growing diameters (between 80-100%).

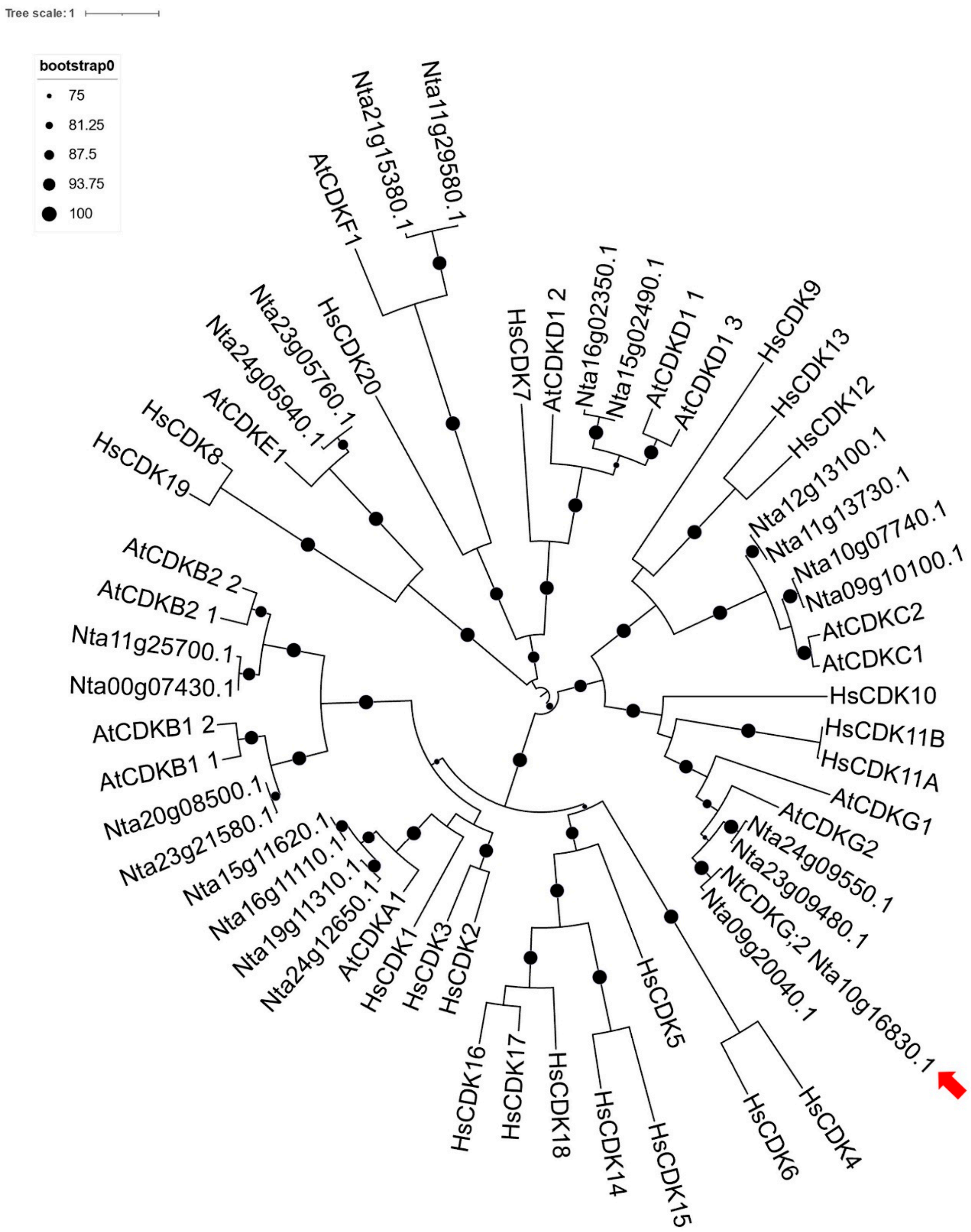

Supplemental Figure S2: Maximum-likelihood tree of CDK sequences from *H. sapiens*, *A. thaliana*, and *N. tabacum*. Bootstrap values are represented as black circles of growing diameters (between 80-100%). The red arrow highlights the NtCDKG;2 used in this study.

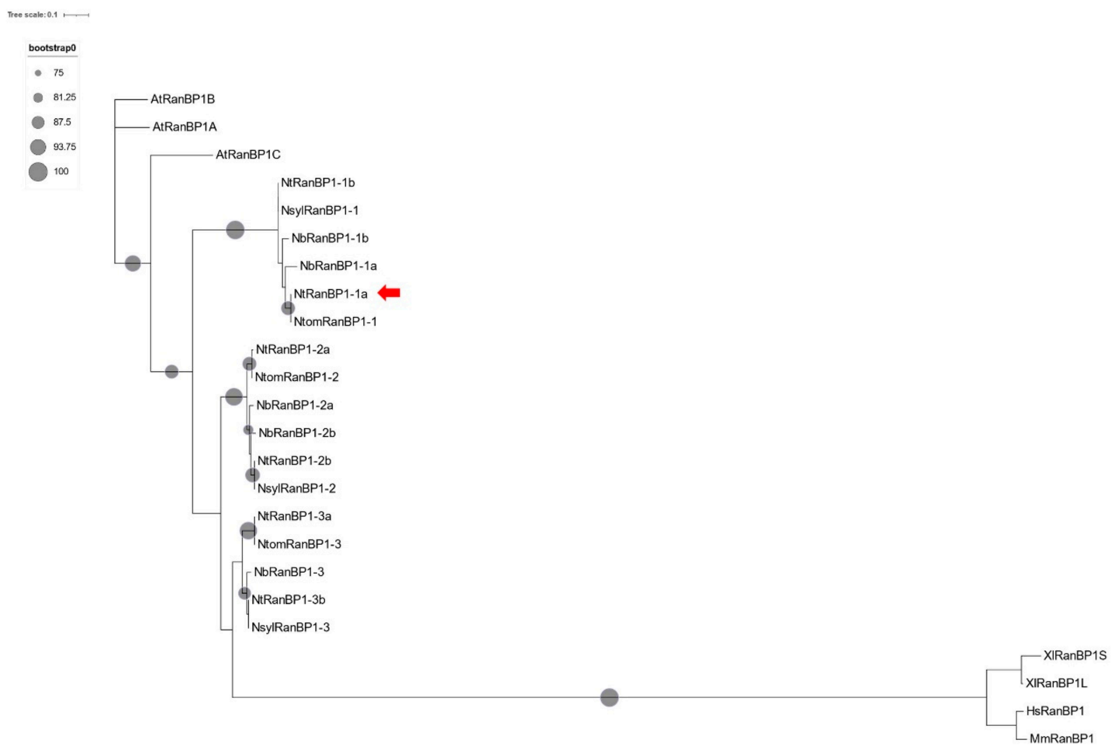

Supplemental Figure S3: Maximum-likelihood tree of Ran binding protein-containing sequences from *A. thaliana* (At), *N. tabacum* (Nt), *N. sylvestris* (Nsyl), *N. tomentosiformis* (Ntom), *N. benthamiana* (Nb), *H. sapiens* (Hs), *M. musculus* (Mm), and *X. laevis* (Xl). Bootstrap values are represented as black circles of growing diameters (between 80-100%). The red arrow highlights the NtRanBP1 used in this study.

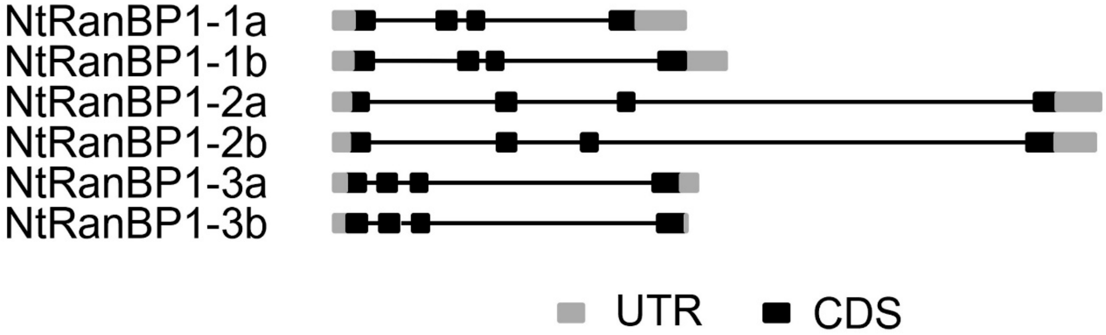

Supplemental Figure S4: Scheme of the gene structures encoding *N. tabacum* RanBP1s. The structure of NtRanBP1 genes is similar; they all are composed of 4 exons and 3 introns. The highest similarity is found between RanBP1s of the same group. Filled boxes are exons, coding sequences (CDS) are represented in black boxes, and thin lines are introns. Untranslated regions (UTRS) are indicated by filled grey boxes. NtRanBP1-1a (LOC107809165), NtRanBP1-1b (LOC107771336), NtRanBP1-2a (LOC107803309), NtRanBP1-2b (LOC107813247), NtRanBP1-3 (LOC107831968), NtRanBP1-3 like (LOC107814775).

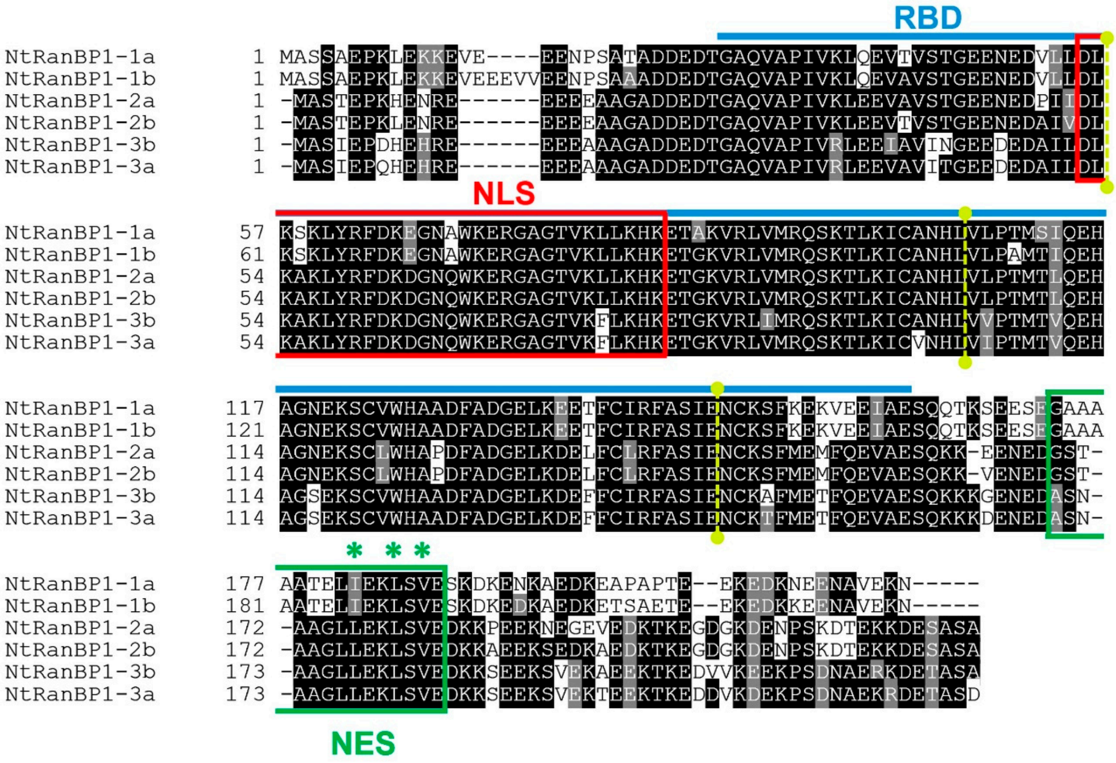

Supplemental Figure S5: Alignment of the six RanBP1 amino acid sequences encoded in the *N. tabacum* genome. The four exons are separated by yellow dashed lines. RBD (indicated by blue line) and NES (highlighted in green) are the most conserved regions, with 100% similarity between the crucial amino acids for NES functionality described by HAASEN et al. (1999) [35] (green asterisks). The importin α-dependent nuclear localization signal (NLS) is highlighted in red. The C-terminal region of the RanBP1 sequences shown the largest protein discrepancy. NtRanBP1-1a (XP_016489244.1), NtRanBP1-1b (XP_016446173.1), NtRanBP1-2a (XP_016482484.1), NtRanBP1-2b (XP_016493973.1), NtRanBP1-3a (XP_016515253.1), NtRanBP1-3b (XP_016495724.1).

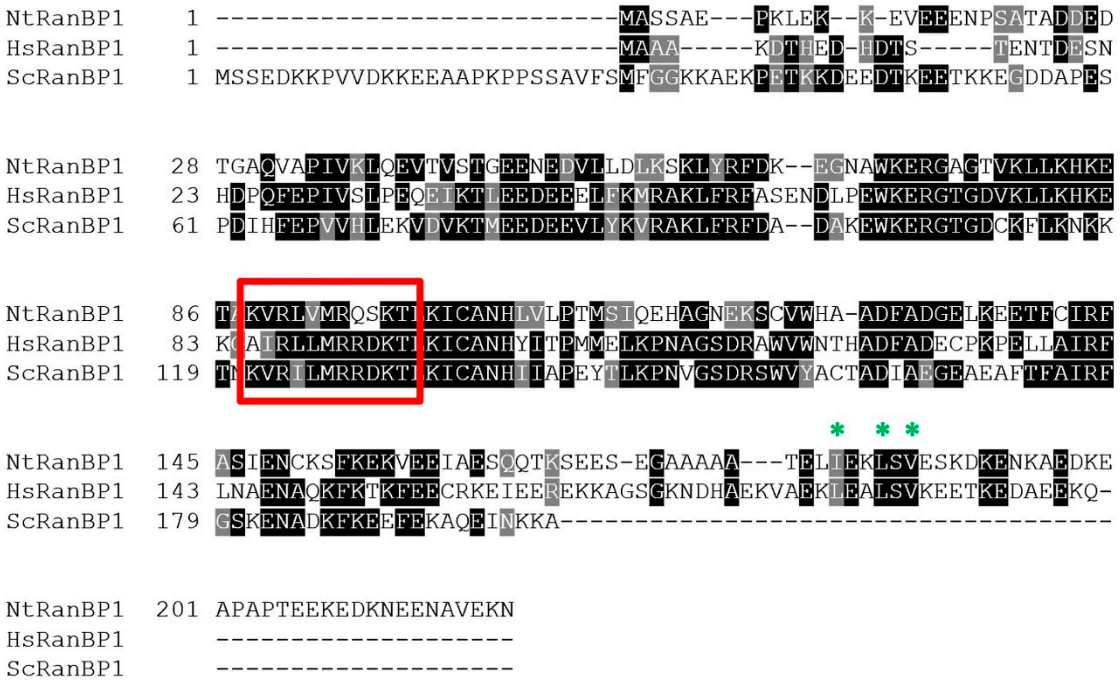

Supplemental Figure S6: Alignment of the RanBP1 amino acid sequences of NtRanBP1, used in this study, and the sequences of *Homo sapiens* and *Saccharomyces cerevisiae* counterparts. The analysis shows that the amino acid residues necessary for nuclear import of the RanBP1 homolog in *S. cerevisiae* (KVRILMRRDKT) are highly conserved in *N. tabacum* and *H. sapiens* (red rectangle). Meanwhile, the crucial amino acids for NES functionality in mammals [35] are absent in *S. cerevisiae* but present in *N. tabacum* (indicated by green asterisks).

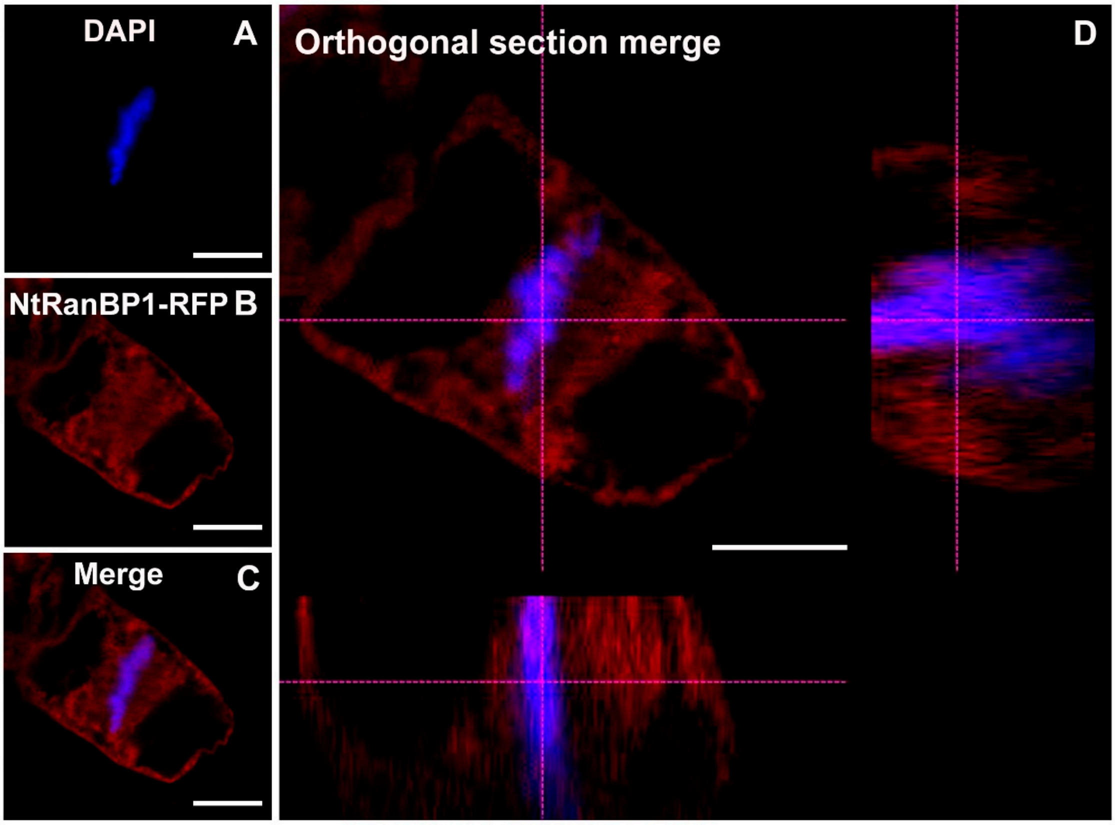

Supplemental Figure S7: Localization of NtRanBP1-RFP in BY-2 cells. During metaphase, the protein is close to chromosomes (B and C) with minimal co-localization to chromatin (D). A: DAPI visualization; B: Visualization of NtRanBP1-RFP; C: overlap of the two previous channels; D: orthogonal section merge. The images of BY-2 cells stably transformed with P35S::NtRanBP1-RFP were obtained with confocal microscope Leica TCS SP5 (Leica Microsystems). Bar = 5 µm.
